# Concept and methodology of the Health Behaviour in School-aged Children (HBSC) study – Insights into the current 2022 survey and trends in Germany

**DOI:** 10.25646/11878

**Published:** 2024-03-04

**Authors:** Kristina Winter, Irene Moor, Jenny Markert, Ludwig Bilz, Jens Bucksch, Kevin Dadaczynski, Saskia M. Fischer, Ronja M. Helmchen, Anne Kaman, Juliane Möckel, Katharina Rathmann, Ulrike Ravens-Sieberer, Franziska Reiß, Theresa Schierl, Raphael Schütz, Saskia Sendatzki, Elisabeth Stürmer, Gorden Sudeck, Matthias Richter, for the HBSC Study Group Germany

**Affiliations:** 1 Martin Luther University Halle-Wittenberg, Halle (Saale); 2 Hochschule Nordhausen – University of Applied Sciences; 3 Brandenburg University of Technology Cottbus-Senftenberg; 4 Heidelberg University of Education; 5 Fulda University of Applied Sciences, Department of Health Sciences; 6 Fulda University of Applied Sciences, Public Health Centre Fulda; 7 Leuphana University Lueneburg; 8 University Medical Center Hamburg-Eppendorf; 9 Technical University Munich; 10 University of Tübingen, Institute of Sports Science; 11 University of Tübingen, Interfaculty Research Institute for Sports and Physical Activity

**Keywords:** CHILDREN, ADOLESCENTS, STUDENTS, HEALTH MONITORING, HBSC, TRENDS, PREVALENCES, SCHOOLS, SURVEY, CROSS-SECTIONAL STUDY, GERMANY

## Abstract

**Background:**

Health Behaviour in School-aged Children (HBSC) is one of the largest international studies on child and adolescent health and cooperates with the World Health Organization (WHO). In Germany, adolescents aged 11, 13 and 15 are surveyed every four years about their health, health behaviour and social conditions. This article describes the HBSC study and in particular the methodology of the current 2022 survey and prior surveys conducted between 2009/10 and 2017/18.

**Method:**

174 schools with a total of 6,475 students participated in the 2022 survey. The survey was conducted using questionnaires and covered a wide range of topics (including mental health, physical activity, bullying experiences, social determinants of health and experiences related to COVID-19). The 2022 survey was complemented by a school principal survey (N = 160). In addition to the current sample, the samples of the three previous surveys with representative data for Germany are presented: 2009/10 (N = 5,005), 2013/14 (N = 5,961) and 2017/18 (N = 4,347).

**Discussion:**

The health of children and adolescents is of great public health importance. The HBSC study makes a substantial contribution by providing internationally comparable results, analysing trends, and providing stakeholders with comprehensive and representative health monitoring data.

## 1. Introduction

### 1.1 Importance of child and adolescent health

Childhood and adolescence represent a critical period in the life course, during which young people are confronted with many simultaneous biological, psychological, cognitive, emotional and social processes of change, with which they cope to varying degrees [1–4]. With increasing age and the onset of puberty, the peer group gradually begin to take a more important role than the family, and adolescents act more autonomously. Various health-related behaviours are initiated that often continue into adulthood. Substance use (e.g. alcohol, tobacco or cannabis), physical inactivity or bullying and violence pose risks not only to adolescents’ current health and well-being but also to their future health [5–9]. In addition, recent research highlights the importance of evidence on mental health during these critical developmental years, not least in times of crisis such as the COVID-19 pandemic. For example, a recent study shows that more than half of mental disorders in adulthood occur before or during adolescence [10, 11]. Thus, the foundations for future health are laid in childhood and adolescence, underscoring the particular importance of these life stages for prevention and health promotion [12–14].

Recent comprehensive data on the health of children and adolescents are of great public health relevance for science, policy and practice, which has taken on a new dimension, especially in times of crisis such as the COVID-19 pandemic [15, 16]. The Health Behaviour in School-aged Children (HBSC) study makes a fundamental contribution to this by providing internationally comparable data, making it possible to map trends and providing decision-makers in the field of child and adolescent health with representative health monitoring data [17]. This article gives a general overview of the HBSC study design. Thus, it presents the methodology of the current survey 2022 for Germany, also taking into account the previous surveys from 2009/10 to 2017/18. Therewith the paper provides the methodological foundation for all other HBSC articles in this issue of the Journal of Health Monitoring.

**HBSC 2022**

**Data holder:** HBSC Study Group Germany

**Objective:** The aim of the study is to analyse the health and health behaviour of students. Continuous health monitoring through the HBSC study contributes to informing decision-makers in policy and practice about the current fields in prevention and health promotion in childhood and adolescence. A particular focus is on the influencing factors and the social contexts of health in the young generation.

**Study design:** Cross-sectional survey by written questionnaire every four years

**Population:** Students with average ages 11, 13, and 15

**Sampling:** Observation units are schools and the class groups clustered within them. From the population of all state general education schools in Germany, a cluster sample was drawn. In order to obtain a representative estimate (close to the distribution of the population), school size and the percentage distribution of students were included in the sampling, stratified by school type and federal state (Probability Proportional to Size (PPS) design).

**Data collection period:** March – November 2022

**Sample size:**

**2022:** 6,475 students

**All four survey cycles (2009/10 – 2022):** 21,788 students

**HBSC survey cycles:**

**Included in the articles in this issue of the Journal of Health Monitoring:**

▶ 2009/10
▶ 2013/14
▶ 2017/18
▶ 2022

More information can be found at https://hbsc-germany.de/ (German)

### 1.2 HBSC – one of the world’s largest studies of child and adolescent health

**Figure 1:**
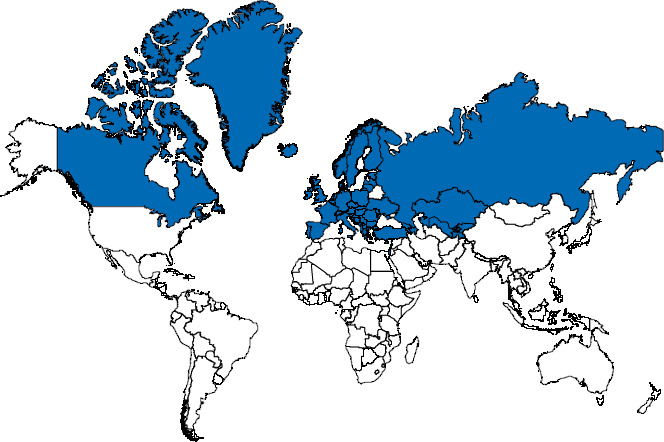
World map of the countries participating in HBSC Source: Own chart

With 51 participating countries (Figure 1), the HBSC study is one of the largest collaborative research projects on child and adolescent health in the world, providing an important national and international resource for monitoring the health of the younger generation. For example, HBSC results are used by the World Health Organization (WHO) for international comparative health reporting on childhood and adolescence [17, 18]. The issues identified by the HBSC study have led to a European strategy on child and adolescent health, with different action, which is available to all WHO Member States as a guide for current and future activities [19]. Recent joint publications by WHO and HBSC present adolescents’ perspectives on the impact of the COVID-19 pandemic on their lives [16].

HBSC aims to collect current data on students’ health and health-related behaviours and to understand how different social determinants affect health and well-being. In addition, there is a specific topical focus for each cycle of the survey. In 2022, for example, the focus was on the impact of the COVID-19 pandemic and adolescent mental health. The results of the HBSC study can be used to inform policies and support developments of health promotion strategies for school-aged children. Therefore, HBSC focuses on understanding the health situation of young people, taking into account social contextual factors and living conditions, and on identifying the need for health-promoting measures [17].

### 1.3 The history of the HBSC study and German participation

**Figure 2:**
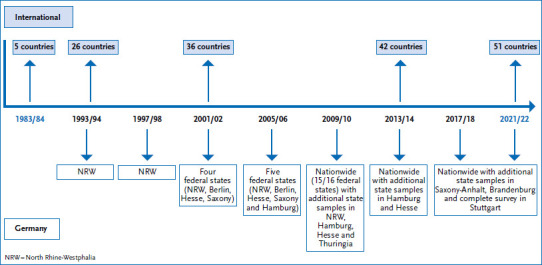
HBSC study: Participating countries and participation of federal states in Germany by survey cycle Source: Own chart

The HBSC study was initiated in 1982 by researchers in England, Finland and Norway. The first survey was carried out in these countries as well as in Austria and Denmark, in 1983/84. Since then, the study has been conducted every four years with a rising number of countries joining the international HBSC network. In the current survey (2022), 51 countries participated with more than 279,000 adolescents [17]. The exchange regarding the topics and methods between the approximately 400 – 500 scientists takes place at regular international meetings and in thematic focus groups, where, among other things, the respective surveys are prepared, the measurement instruments are validated and expanded, and publications are centrally planned. Germany joined the HBSC network with the 1993/94 survey, initially with its most populous state, North Rhine-Westphalia (NRW). Other federal states (Saxony, Hesse, Berlin, Thuringia and Hamburg) were included in the subsequent cycles in 1997/98, 2001/02 and 2005/06. With the exception of Baden-Wuerttemberg, all federal states participated in the 2009/10 cycle. Data from 2009/10 onwards are therefore included in the trend analyses in this issue. Since the follow-up survey in 2013/14, the HBSC study has been conducted every four years in all 16 federal states. In the current survey in 2022, as in the previous cycle in 2017/18, the nationwide survey was conducted with two supplementary state samples in Brandenburg [20] and Saxony-Anhalt [21] as well as a survey for the city of Stuttgart (Figure 2).

**Figure 3:**
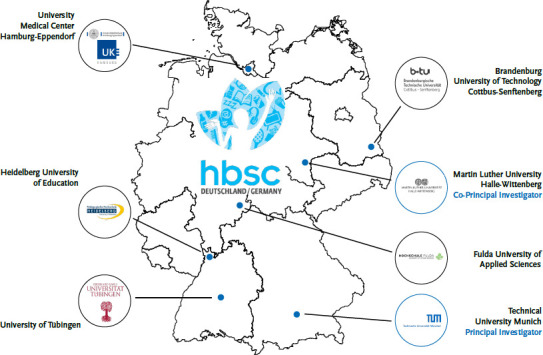
HBSC Study Group Germany (2023) Source: Own chart

All surveys conducted in Germany were carried out under the joint responsibility of the HBSC Study Group Germany. The HBSC Study Group Germany currently consists of seven sites (Figure 3; listed in alphabetical order): Brandenburg University of Technology Cottbus-Senftenberg (Prof. Dr. Ludwig Bilz), Fulda University of Applied Sciences (Prof. Dr. Katharina Rathmann, Prof. Dr. Kevin Dadaczynski), Heidelberg University of Education (Prof. Dr. Jens Bucksch), Martin Luther University Halle-Wittenberg (Dr. Irene Moor; Co-Principal Investigator), Technical University of Munich (Prof. Dr. Matthias Richter; Principal Investigator), University Medical Centre Hamburg-Eppendorf (Prof. Dr. Ulrike Ravens-Sieberer), University of Tübingen (Prof. Dr. Gorden Sudeck). In addition, a total of 11 research associates and 14 student assistants supported the preparation and conduction of the current HBSC survey.

## 2. Methods

### 2.1 Study design and sample design

The Health Behaviour in School-aged Children (HBSC) study is designed as a cross-sectional study that takes place every four years in the school setting and surveys students aged around 11, 13 and 15 (mean deviation of 0.5 years). In Germany, these age groups mainly comprise grades 5, 7, and 9. International HBSC guidelines call for a total national sample of 4,500 students, with a sample size of approximately n = 1,500 per age group [17].

The units of the HBSC study are schools and school classes clustered within them. In preparation, the sample calculation was initially based on the current state-specific school directories of the state school authorities (data basis for the survey: school year 2020/21). The population inculded all state general education schools in Germany.

The distribution of students by federal state, school type, grade and sex was taken into account in the calculation. This means that federal states with a higher population of students in the respective grades (e.g., Bavaria) were included to a higher extent than those with a lower population sample. Based on the experience of previous HBSC cycles, a downward trend in willingness to participate can be observed for HBSC in Germany (see 3. Results on representativeness and response rates). As a lower willingness to participate was also expected for the 2022 survey, e.g. due to challenges in schools related to the COVID-19 pandemic, a response rate of 10 % at school level and 50 % at student level was assumed for the sample calculation. As each data form all countries will be cleaned as part of the international data cleaning process, especially regarding the comparability of the targeted age groups (adjusting the age difference of +/- 0.5 years to the target population), an additional quality-neutral exclusion of 20 % of the sample was taken into account for the sample size calculation. Based on this calculation, the next step was to draw a random cluster sample. In order to obtain a better estimate (closer to the distribution of the population), the school size and the distribution of students, stratified by school type, were included in the sample (probability proportional to size).

### 2.2 Study content and survey instruments

#### Student survey

The focus of the HBSC study is the collection of health indicators and related social determinants of health in students. The survey is conducted in all participating countries according to standardised and internationally agreed methods, using a questionnaire that adolescents complete themselves using a paper-and-pencil method or fill out online or offline with a tablet. The questionnaire consists of a mandatory section and an optional section, which ensures international comparability while allowing each country to set its own research priorities. The questions used are continuously developed and validated [17]. Most of the instruments (from the mandatory section) used are items in English, of which most of them have been used in previous cycles in Germany and therefore have already been translated. New items included in the 2022 survey were translated into German in a multi-stage process (forward-backward translation). Under the slogan ‘Don’t talk about us without us’, the international HBSC study network (focus group ‘Youth Engagement Advisory Group’) regularly involves young people in identifying current and important issues for them. These results are considered in the respective HBSC cycles [17]. Further information can be found on the international HBSC website [22].

**Figure 4:**
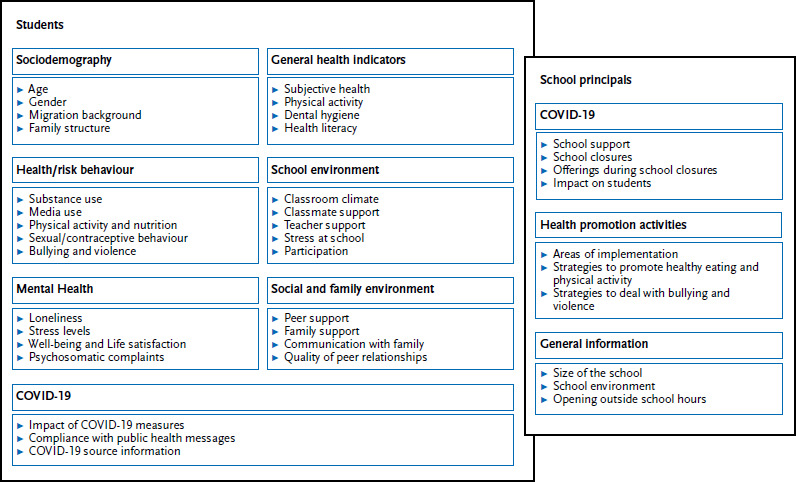
Insight into the subject areas of the 2022 HBSC study Source: Own chart

Detailed information regarding the items and scales used (Figure 4) as well as the methodological procedure and validation can be found in the international research protocol [17].

#### Survey of school principals

There is growing evidence that, in addition to individual factors, institutional and contextual factors at the meso level also play a role in adolescent health. Examples include the type of school, but also organisational, structural, cultural and physical factors of schools and classes, such as school norms and values, class or school size, sociodemographic/economic composition of the students population, equipment, premises or school hours [4, 23]. Alongside the family as a secondary socialisation setting, school is an important context for the psychosocial and health-related development of young people. Not only do children and adolescents spend a large proportion of their time at school [1, 24, 25], but they also interact with their peers and teachers and have a variety of positive (e.g. perceptions of support) and negative (e.g. pressure to perform, fear of failure) experiences. The school context can therefore shape adolescents in many ways and influence their health, making it an important setting for health promotion and prevention [25–28]. For this reason, in addition to the survey of students, a survey of school principals was conducted in the 2022 cycle. The aim was to collect contextual information from schools and to assess the commitment of schools to implementing health-promoting activities. In addition, the restrictions imposed on schools by the COVID-19 containment measures (including restrictions on school operations), but also the opportunities to expand the range of services (support services) were also considered [29]. School principals (or their deputies) were asked to complete an online questionnaire. Figure 4 provides an insight into the different topics covered by the two questionnaires in the current survey.

### 2.3 Pretest

The German questionnaire was pre-tested with regard to processing time, content ambiguities and comprehension questions, with a special focus on the comprehension among younger students. For this purpose, students from grade 5 (n = 21) and 7 (n = 23) from different school types were interviewed between July and August 2021. Due to the COVID-19 pandemic, the pretests were conducted online. Students received the link to the online questionnaire and completed it using the ‘think aloud’ method. All comments made by the participants were transferred to the pretest protocols. The majority of the questions and answer options were easy to answer for the students. However, some students had problems with longer introductions or respondents lacked further answer options. In addition, there were some comprehension difficulties, e.g., on the health literacy scale for younger students. As a result, minor changes were made to the questionnaire (where possible).

### 2.4 Recruitment, implementation and data collection

Permission to conduct the HBSC study in schools was obtained from the relevant ministries or state education authorities in each federal state (with the exception of North Rhine-Westphalia, where schools decide autonomously whether to participate). Approvals were granted in close cooperation with the relevant data protection officers – appropriate data protection policy was part of the approval process and an integral part of the approval to conduct the study. The approval process took up to nine months, depending on the federal state. As recruitment was depending on approval, there were delays in the recruitment and survey process in some federal states. Recruitment of schools was carried out in a decentralised but standardised way by all sites in the study group, with each site taking responsibility for certain federal states. In the first step, the randomly selected schools were invited by post and e-mail to participate in the survey. In addition to the letter of invitation, the schools received information material about the study. If schools did not respond within two weeks, they were contacted by telephone and invited to participate. The telephone contact proved to be particularly beneficial, although it was also very resource-intense [30, 31]. Schools that agreed to participate were closely accompanied and supported by the HBSC recruitment team and kept informed of all the steps to be taken.

After accepting to participate in the survey, each school received a comprehensive information pack, including survey materials and flyers, so that school staff, parents and students had all relevant information regardingthe HBSC study at an early stage. In addition, key information was made available on the study’s password-protected website for each target group.

In each school, students in class groups of one class each in grade 5, 7 and 9 were surveyed. In some cases (e.g. at the request of the schools or in case of lower participation rates in the respective federal states or school types) more than one class per grade was included in the survey. The participating classes were selected randomly by the school principal. The survey could be completed online, offline via tablets, or by printed questionnaires, depending on the technical infrastructure and preferences of the school. The questionnaire could only be completed if a parent/guardian and the students themselves (from grade 7) had given their consent to participate in the survey. In order to ensure a standardised survey procedure, detailed information and instructions were provided to school staff well in advance of the survey. For example, to gain access to the online questionnaires, a list of access codes was prepared in advance and provided to each class individually. These were only used to access the survey and to monitor the respective grade, school type and federal state. Upon completion of the survey, the data were re-encrypted. If schools opted for an offline survey using tablets (this option was only available in Brandenburg), the survey was administered by members of the research team on site at the schools. All data were collected anonymously.

Irrespective of the mode of data collection, it is nearly impossible to identify individual students from the data. Due to the recording of the data, it is also no longer possible to identify individual schools. The representative federal state samples are an exception, as each participating school agreed on receiving a school-specific feedback (school health profiles) as an incentive. However, identification was also stored separately and is subject to strict data protection guidelines.

All participating schools received an individual certificate as an incentive for taking part in the HBSC survey. Schools also receive a summary of the latest results from the HBSC study Germany.

The survey period ranged from March to November 2022. After the survey was completed, all data collected in the form of anonymous printed questionnaires was sent to an external data provider for data entry. The Brandenburg University of Technology Cottbus-Senftenberg used the tool LimeSurvey to collect data from questionnaires completed offline on tablets or online. The data collected was stored directly in the tool and created as a dataset. Finally, all data was merged into one overall dataset followed by an internal review and quality adjustment. Following this, the international HBSC study network undertook a central cleansing for all participating countries to ensure international comparability [17].

## 3. Results on representativeness and response rates

### 3.1 Sample and response rates over time

**Figure 5:**
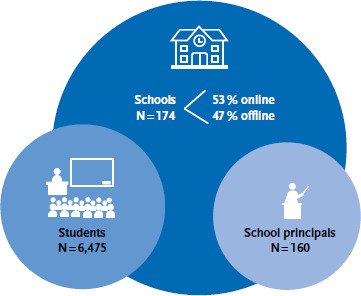
Overview of the realised HBSC 2022 sample Source: Own chart

A total of 174 schools with a total of 7,935 students (unadjusted net sample) participated in the 2022 national HBSC cycle. More than half of all participating schools chose to fill out the questionnaire online instead of paper-and-pencil. As part of the internationally standardised data cleaning by the Data Management Centre in Bergen/Norway, quality-neutral omissions in the data set were corrected. These mainly include deviations in the age groups, where the variance exceeds +/- 0.5 years, as well as implausible outliers in the response data (e.g., height and weight). For these reasons, a total of 1,478 cases (18.6 %) were excluded. The realised sample (net), which was used as the data basis for the analyses, therefore consists of N = 6,475 students. In addition, 160 school principals were surveyed (Figure 5).

Table 1 provides information on the sample distribution of schools and students participating in the 2022 national survey in each federal state and in total. The sample realised in most of the federal states corresponds approximately (partly with slight deviations) to the proportional targets. Any deviations are compensated by weighting the data for subsequent analyses (see 3.2 Weighting).

Table 2 shows the realised samples for the survey years 2009/10 to 2022 by age and gender. Overall, the participation rate is broadly similar for girls and boys and by age group. One exception is the case of gender diverse adolescents, for whom it is not possible to make any statements about trends, as this third category was added as a response option for the first time in the current cycle. In total, more than 100 young people identified themselves as gender diverse.

The response rate decreased over the last twelve years at both student and school level. While about half of the schools and 86 % of the students participated in the 2009/10 survey cycle, only 8.6 % of the schools and just over half of the students participated in the current survey. Detailed information on the survey cycles 2009/10 to 2017/18 can be found in the previous methodological articles [32–34].

### 3.2 Weighting

In order to achieve a representative sample based on the distribution of students in Germany, data from the actual and target samples were compared and checked for their distribution at the level of federal states and school types. Deviations in the realised sample are due, among other things, to a lower willingness to participate and to data adjustments. Deviations from the official statistics (school year 2020/21) were taken into account by means of a weighting variable. This is a standardised methodological procedure to counteract discrepancies in the response rate and thus distortions in the comparative values (e.g. disproportionality of one type of school) [35]. For age and (binary) gender, proportionally equal proportions were preferred. As the gender category ‘diverse’ was included for the first time in 2022, but representative distributions for this age group are not yet available in Germany, the corresponding sample case numbers were used as an estimate for the population. Thus, 49.2 % of girls and 49.2 % of boys and 1.7 % of diverse adolescents were included in the sample. The weighting in the HBSC 2022 includes an equalisation of the data by federal state, school type, gender and age group. The weighting variable is used in all analyses in exception of absolute figures which are presented unweighted.

## 4. Discussion

The HBSC study provides valid and representative data on the health of children and adolescents in Germany, which are internationally comparable and can be monitored over time. From a public health perspective, HBSC is therefore an important source of data for health monitoring and health reporting. One of the most important goals of HBSC is to provide a broad database for health policy decisions. In addition to contributing to international and national health reporting and to the development of health goals, the regional HBSC data of the federal states of Saxony-Anhalt and Brandenburg have been used to identify specific fields of action. These analyses at the regional level [20, 21] have led to the initiation of health-promoting measures, for example a school-based intervention in Saxony-Anhalt to promote the mental health of adolescents. In some countries, the HBSC study is the only source of data on the health of children and adolescents, and in Germany, too, the HBSC was one of the first studies to report comprehensively on the health of this age group, alongside the KiGGS study [36].

This paper provides an insight into the origins and development of the HBSC study in Germany and presents the methods and study design of the current 2022 survey. In addition to the complete survey process, the current case numbers and response rates are presented. With a focus on trend analysis, case numbers and response rates from the previous twelve years of the survey were also used for comparison. This publication serves as a basis for the thematic contributions in this issue, which deal with subjective health and psychosomatic complaints (Reiß & Behn et al. [37]), physical activity (Bucksch et al. [38]), health literacy (Sendatzki & Helmchen et al. [39]), bullying (Fischer et al. [40]) and health inequalities (Moor et al. [41]). These papers will map the health situation of children and adolescents in Germany and track trends over the last twelve years (2009/10 – 2022). They provide information on current challenges as well as positive developments and are therefore an important source of information for stakeholders in the field of child and adolescent health.

### 4.1 Strengths and weaknesses of the HBSC study

The strengths of the HBSC study are manifold: in particular, the international comparability of 51 countries, the possibility of analysing trends over time and the consideration of social determinants and contextual factors. The measurement instruments are valid and the data are representative of children and adolescents in the age groups covered in Germany. The current 2022 survey also provides a special opportunity to examine the health situation of adolescents considering the possible effects of the COVID-19 pandemic and to compare it with the situation before the pandemic (2017/18 survey). In addition to the survey of students, it was also possible to conduct a survey of school principals, which made it possible to include additional assessments of the structural conditions of the school context. In the current survey, it was also possible to achieve a more differentiated survey of gender identity by adding the category ‘diverse’. This means that the HBSC study can provide data on a minoritised group in this age group for the first time at the national level; data on gender diverse (young) adults can be found, for example, in the study ‘German Health Update’ (GEDA) [42]. Insights into the health situation of gender diverse adolescents can be found in the different publications in this issue. These findings are of great importance, especially in view of the research gaps. However, the number of cases is sometimes too small for all statistical analyses. In addition, the response category ‘diverse’ does not cover gender diversity in its entirety, but serves as a collective term that does not allow for further differentiation [42].

The broad range of health topics are a strength and a limitation at the same time. Although this has the advantage that a wide range of different health-related topics can be continuously surveyed, they are only touched on in passing. The HBSC study can therefore provide indications that can be analysed in more detail in further research projects (e.g., in focus group interviews or topic-specific studies). In addition, HBSC is designed as a cross-sectional study. Although this makes it possible to analyse current prevalence and trends, it is not possible to deduce causalities, only to illustrate correlations. Another limitation and challenge for most scientific studies is the declining willingness to participate in the surveys [30], which is a very alarming development for science. It is expected that the willingness of both schools and students to participate will continue to decline in the coming years. Without the willingness of study participants, reliable results cannot be obtained. Although the response rate for the HBSC study was similar to other surveys of this age group [43], incentive structures are essential to increase the response rate. These need to be implemented at every stage of the project, from planning (e.g., adequate staffing, good coordination), through contact initiation (e.g., personal contact, relevance of topic, special attention to privacy), implementation (e.g., all costs covered, close contact), to completion of the study (feedback of results) [31, 44]. Although the HBSC study is self-funded, these aspects have been implemented as far as possible by the HBSC Study Group Germany. In addition, willingness to participate is inextricably linked to staff shortages in schools. The assumption that online surveys would achieve a higher willingness to participate with a lower use of resources [34] could not be confirmed with HBSC. Rather, it was found that schools were still lagging behind in terms of digitisation and preferred a printed survey. Further, the online questionnaire was more likely to be abandoned than the paper questionnaire.

An additional challenge is the approval process for the study, which any health study in a school context is faced with. The sometimes state-specific adaptations and the fulfilment of specific requirements, which are seen as a prerequisite for approval, require many feedback loops in the preparation of the study (both within the study network and between the study coordinator and the responsible ministries/state education authorities). If access would be easier – especially for regularly recurring studies such as HBSC – this could reduce the workload on both sides and also counteract delays in the study process (e.g., due to long waiting times in the approval procedure). This would also make it possible to significantly reduce the duration of the study recruitment.

### 4.2 Conclusion

The next generation is the foundation of our future society. Investing in healthy development is therefore an important public health objective. There is evidence that social crises such as the COVID-19 pandemic have a massive impact (beyond the infectious disease) on the living environment and health situation of children and adolescents, thus emphasising the relevance of child and adolescent health research [4, 45, 46]. Regular health monitoring is needed to assess the health impact on the young generation and to identify options for action. This requires close cooperation between all stakeholders in policy, practice and research, and of course with children and adolescents themselves, in order to give them the best possible opportunities to grow up healthy.

## Key statement

- HBSC is one of the largest surveys of child and adolescent health in the world.
- 51 countries with more than 279,000 adolescents participated in the current 2022 survey.
- In Germany, a total of 174 schools (response rate: 8.4 %) with 6,475 students (response rate: 56.8 %) and 160 school principals was taken into account in the 2022 survey year.
- For Germany, data from twelve years (2009/10 to 2022) with 21,788 students are used for trend analysis.
- The HBSC provides continuous and comprehensive internationally comparable data on the health of children and adolescents and enables trend analysis.

## Figures and Tables

**Table 1:** Distribution of the adjusted unweighted total sample of HBSC 2022 by federal state Source: HBSC Germany 2022

Federal state	Number of participating schools	Realised number of students (ACTUAL) (after adjustment)	ACTUAL Proportion of students	TARGET Proportion of students
Baden-Wuerttemberg	15	556	8.6 %	13.7 %
Bavaria	26	929	14.3 %	15.5 %
Berlin	13	210	3.2 %	4.1 %
Brandenburg	10	434	6.7 %	3.0 %
Bremen	1	40	0.6 %	0.8 %
Hamburg	3	100	1.5 %	2.2 %
Hesse	23	936	14.5 %	7.7 %
Mecklenburg-Western Pomerania	5	156	2.4 %	1.8 %
Lower Saxony	23	798	12.3 %	10.3 %
North Rhine-Westphalia	16	642	9.9 %	22.1 %
Rhineland-Palatinate	7	260	4.0 %	4.8 %
Saarland	2	63	1.0 %	1.1 %
Saxony	10	408	6.3 %	4.4 %
Saxony-Anhalt	5	370	5.7 %	2.3 %
Schleswig-Holstein	6	250	3.9 %	3.8 %
Thuringia	9	323	5.0 %	2.5 %
**Total**	**174**	**6,475**	**100 %**	**100 %**

**Table 2:** Case numbers, percentage and response rates of the HBSC study over the last twelve years by gender and age group^*^ Source: HBSC Germany 2022

	2009/10		2013/14		2017/18		2022		Total	
	N	%	N	%	N	%	N	%	N	in %
**Gender**										
Female	2,576	51.5	2,926	49.1	2,306	53.0	3,258	50.3	11,066	51.0
Male	2,429	48.5	3,035	50.9	2,041	47.0	3,074	47.5	10,579	48.5
Gender diverse	–	–	–	–	–	–	112	1.7		
**Age group**										
11-year-olds (5th grade)	1,687	34.0	1,736	29.4	1,387	32.2	2,132	33.3	6,942	32.2
13-year-olds (7th grade)	1,628	32.9	2,070	35.0	1,403	32.6	2,160	33.7	7,261	33.6
15-year-olds (9th grade)	1,640	33.1	2,104	35.6	1,515	35.2	2,113	33.0	7,372	34.2
**Total**	RR		RR		RR		RR		RR	
Students	5,005	86.0	5,961	72.5	4,347	52.7	6,475	56.8	21,788	67.0
Schools	187	48.0	188	24.4	146	15.6	174	8.4	508	24.1

^*^Absolute figures unweighted, percentage figures weighted

Some of the data still contain missing values in individual variables and survey years, which is why there may be deviations in the total number of cases

RR = response rate
